# Analysis of the microbial spectrum of urinary tract infections and antibiotic resistance of *UPEC* in Central Inner Mongolia, China

**DOI:** 10.3389/fmicb.2025.1599902

**Published:** 2025-09-24

**Authors:** Jingru Zhang, Shengyuan Wen, Liqing Chen, Jianxiong Qin, Ziling Liu

**Affiliations:** ^1^The Clinical Laboratory of Baotou Central Hospital, Baotou, China; ^2^Baotou Medicine College, Inner Mongolia University of Science and Technology, Baotou, China

**Keywords:** urinary tract infections, *Escherichia coli*, antibiotic resistance, quinolone resistance genes, levofloxacin

## Abstract

Urinary Tract Infections (UTIs) remain a significant global health concern, with the pathogen spectrum and antibiotic resistance patterns critically influencing clinical management. This study aimed to analyze the microbial profile of UTIs from 2022 to 2024, focusing on *Escherichia coli* (*E. coli*), the predominant pathogen, by evaluating its antibiotic resistance phenotypes, biofilm formation capability, and mutations in *GyrA, ParC* and Plasmid-Mediated Quinolone Resistance (PMQR) genes prevalence. Midstream urine samples from UTI patients were collected and analyzed for microbial identification. *E. coli* isolates were tested for antibiotic susceptibility, particularly to levofloxacin, the most frequently used antibiotic in our hospital. Biofilm formation was assessed, and mutations in *GyrA, ParC* and PMQR genes were sequenced to determine resistance mechanisms.The pathogen spectrum revealed that *Enterobacteriaceae* were the most prevalent (44.42%), with *E. coli* being the dominant species. Over 70% of *E. coli* isolates exhibited resistance to levofloxacin, and 58.97% (46/78) demonstrated biofilm-forming ability. Among levofloxacin-resistant strains, 75.64% (59/78) showed high-level resistance (MIC ≥ 8μg/mL). The most common mutations in *GyrA* were Ser83Leu (89.74%) and Asp87Asn (71.79%), while Ser80Ile (74.36%) was predominant in *ParC*. PMQR genes were detected in 17.95% (14/78) of isolates. The elevated prevalence of quinolone-resistant *E. coli* in urinary tract infections within this region, combined with intricate resistance gene mutations and generally strong biofilm-forming capabilities, underscores the critical necessity for rational antibiotic stewardship.

## 1 Introduction

Urinary Tract Infections (UTIs) are among the most prevalent bacterial infections globally, affecting millions of individuals annually and imposing a substantial burden on healthcare systems (Ioana et al., 2022). Despite advancements in medical science, the incidence of UTIs remains alarmingly high, particularly due to the emergence of multidrug-resistant pathogens. *Escherichia coli* (*E. coli*) is the predominant causative agent of UTIs, commonly referred to as *Uropathogenic Escherichia coli* (*UPEC*), accounting for approximately 70-95% of community-acquired and 50% of hospital-acquired cases. However, the increasing prevalence of antibiotic resistance, particularly to fluoroquinolones, has significantly complicated the clinical management of these infections. The widespread and often indiscriminate use of antibiotics, such as levofloxacin, has led to the development of resistance mechanisms in *E. coli*. These mechanisms include mutations in the Quinolone Resistance-Determining Region (QRDR) genes like *GyrA* and *ParC*, as well as the acquisition of PMQR (Hu et al., 2025). Additionally, the ability of *E. coli* to form biofilms further exacerbates its resistance to antibiotics, as biofilms provide a protective barrier against antimicrobial agents and host immune responses.

Understanding the microbial profile of UTIs and the resistance patterns of predominant pathogens is crucial for guiding empirical antibiotic therapy and improving patient outcomes. In this study, we aimed to analyze the microbial spectrum of UTIs in our region from 2022 to 2024, with a particular focus on *E. coli*. We evaluated its antibiotic resistance phenotypes, biofilm-forming capability, and mutations in key quinolone resistance genes. Our findings highlight the urgent need for rational antibiotic use and the implementation of effective infection control measures to combat the growing threat of antibiotic-resistant UTIs.

This study contributes to the existing scope of knowledge by providing detailed insights into the resistance mechanisms in fluoroquinolones of *E. coli* in UTIs, emphasizing the interplay between biofilm formation, genetic mutations, and antibiotic resistance. The results underscore the importance of antimicrobial stewardship and the development of novel therapeutic strategies to address the challenges posed by resistant pathogens.

## 2 Methods

**Figure 1:**
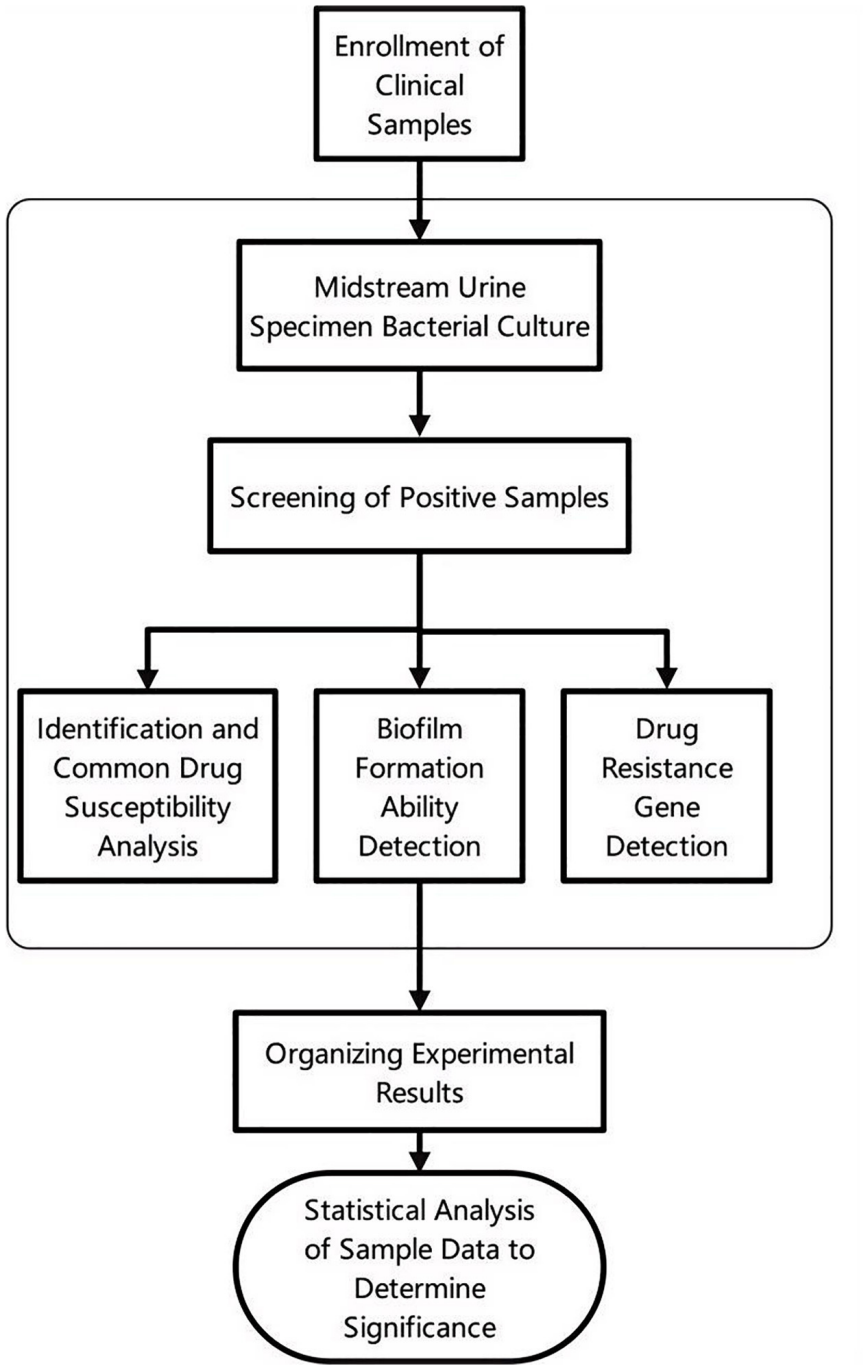
The flowchart of the experimental procedure.

The entire experimental process is illustrated in Figure 1, which provides a detailed visual representation of the key steps involved in the study.

### 2.1 Bacterial strain collection

This study screened nine thousand three hundred and twelve patients diagnosed with Urinary Tract Infections (UTIs) at Baotou Central Hospital from 2022 to 2024. A urinary tract infection is defined as an inflammatory condition caused by pathogenic microorganisms invading the urinary system, including the kidneys, ureters, bladder, and urethra. Depending on the site of infection, UTIs can be classified as upper urinary tract infections or lower urinary tract infections. The inclusion criteria for patients were as follows: Clinical diagnosis of UTI accompanied by typical symptoms. Positive midstream urine culture results with a bacterial colony count of ≥10^7^ CFU/L (Bilsen et al., 2024; Chan et al., 2025). Exclusion of patients with non-infectious urinary tract diseases or contaminated samples. Midstream urine specimens were collected from eligible patients for pathogen isolation and identification. All samples were processed in accordance with hospital infection control standards and laboratory protocols to ensure data accuracy and reliability.

### 2.2 Bacterial identification and antimicrobial susceptibility testing

The midstream urine specimens collected from patients were cultured on sheep blood agar plates under standard laboratory conditions. Specimens with a bacterial colony count of ≥10^7^ CFU/L after incubation were selected for further analysis. Pure bacterial colonies were obtained through sub culturing and streaking techniques to ensure isolate purity.

For bacterial identification and antimicrobial susceptibility testing using the Clinical and Laboratory Standards Institute (CLSI, 2021) guidelines, the pure cultures were processed using the automated microbial identification and susceptibility analysis system (BD Phoenix™, Becton Dickinson, U.S.A.). This system employs advanced biochemical and enzymatic assays to accurately identify bacterial species and determine their susceptibility profiles to a panel of antibiotics.

### 2.3 Drug resistance gene amplification and sequencing

The Polymerase Chain Reaction (PCR) was employed to amplify quinolone resistance-related genes, including the QRDR genes (*GyrA, ParC*) and PMQR genes (*qnrA, qnrB, qnrD, qnrS, qepA, aac(6*′*)-Ib-cr*), using polymerase chain reaction (PCR) (Josefina et al., 2025; Wajeeorn et al., 2025). The primer sequences and their corresponding annealing temperatures are presented in Table 1. After the PCR products were subjected to agarose gel electrophoresis, the gel imaging system was used to observe whether the target bands were successfully amplified. Following the amplification of QRDR genes, sequencing was performed to identify specific resistance mutation sites. MEGA7 software was used to align the corresponding sequences of the sequenced sequences with those of *E. coli* standard strain (*K12*), and the mutation sites of nucleotides and amino acids were analyzed.

**Table 1:** Antibiotic resistance genes and its corresponding primer sequence.

**Gene**	**Primer sequence (5^′^-3^′^)**	**Fragment size (bp)**	**Annealing temperature (°C)**
*GyrA*	F:GACCTTGCGAGAGAAATTACAC	540	59
	R:GATGTTGGTTGCCATACCTACG		
*ParC*	F:CGGAAAACGCCTACTTAAACTA	446	59
	R:GTGCCGTTAAGCAAAATGT		
*qnrA*	F:TCAGCAAGAGGATTTCTCA	674	59
	R:GGCAGCACTATTACTCCCA		
*qnrB*	F:GATCGTGAAAGCCAGAAAGG	469	59
	R:ACGATGCCTGGTAGTTGTCC		
*qnrS*	F:ACGACATTCGTCAACTGCAA	417	59
	R:TAAATTGGCACCCTGTAGGC		
*qnrD*	F:CGAGATCAATTTACGGGGAATA	582	59
	R:AACAAGCTGAAGCGCCTG		
*qepA*	F:GCAGGTCCAGCAGCGGGTAG	218	59
	R:CTTCCTGCCCGAGTATCGTG		
*aac(6′)-Ib-cr*	F:TTGCGATGCTCTATGAGTGGCTA	482	59
	R:CTCGAATGCCTGGCGTGTTT		

### 2.4 Quantitative assessment of *E. coli* biofilm formation

The crystal violet staining method was employed to quantitatively assess biofilm formation according to the detection methods previously established. The 96-well plate was incubated at 35 °C for 72 h to observe bacterial growth. Negative control wells contained uninoculated sterile broth only. The absorbance of each well was measured at a wavelength of 570 nm using a microplate reader. The biofilm-forming ability was determined based on the absorbance values (OD) as follows:non-adherent (OD ≤ OD_C_), weakly adherent (OD_C_ < OD ≤ 2 × OD_C_), moderately adherent (2 × OD_C_ < OD ≤ 4 × OD_C_), strongly adherent (OD>4 × OD_C_) (Ratajczak et al., 2021). Here, OD_C_ is defined as three times the standard deviation of the average absorbance of the three negative control wells, and OD represents the average absorbance of each bacterial strain.

### 2.5 MIC determination of levofloxacin against *UPEC*

The MIC (Minimum Inhibitory Concentration) of levofloxacin, a commonly used quinolone antibiotic for *UPEC* treatment in the region, was determined for 78 randomly selected *UPEC* strains. The broth microdilution method was employed to prepare a series of drug concentrations: 1024, 512, 256, 128, 64, 32, 16, 8, and 4 μg/mL. The standard MIC detection protocol (CLSI, 2012) was conducted to obtain the MIC value for each strain. The high-level resistance threshold of *UPEC* to levofloxacin is defined as ≥8 μg/mL.

### 2.6 Analysis of statistical data

Statistical analysis was performed using SPSS 26.0. The categorical data were presented as frequencies or percentages. Venn diagrams were employed to analyze the correlation between drug-resistant strains and the presence of related drug-resistant genes and gene mutations. The linear correlation between biofilm-forming ability and MIC was assessed using Pearson's correlation coefficient; the association between biofilm-forming ability and mutation count was analyzed via Spearman's rank correlation.

## 3 Results

### 3.1 Microbial spectrum of urinary tract infections

**Figure 2:**
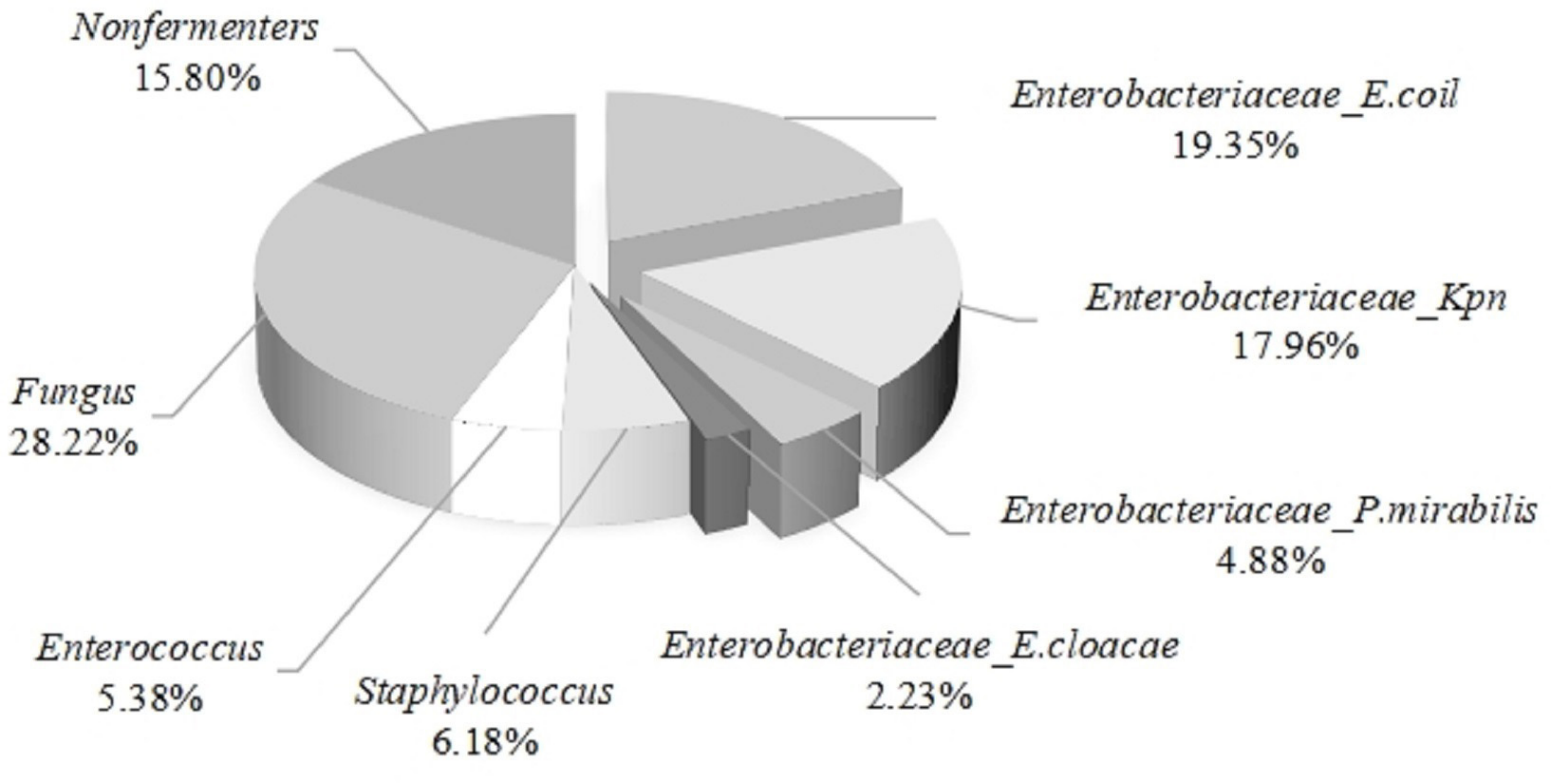
Distribution of 9312 strains of Uropathogens during 2022–2024.

The midstream urine specimens from nine thousand three hundred and twelve patients with urinary tract infections were cultured and identified, revealing the following results: the proportion of *Enterobacteriaceae* infections was 44.42%, followed by fungal infections at 28.22%. The infection rates of non-fermentative bacteria, *Staphylococcus spp*., and *Enterococcus spp*. were 15.80%, 6.18%, and 5.38%, respectively. Among *Enterobacteriaceae* infections, *E. coli* accounted for the highest proportion at 19.35%, followed by *Klebsiella pneumoniae* at 17.96%, while *Proteus mirabilis* and *Enterobacter cloacae* accounted for 4.88% and 2.23%, respectively (see Figure 2). From the above, it can be concluded that *E. coli* is the primary pathogen in urinary tract infections.

### 3.2 The antimicrobial susceptibility results of common antibiotics against *UPEC*

The analysis of the antimicrobial susceptibility results of 1,802 *UPEC* to commonly used antibiotics revealed that carbapenems (imipenem, meropenem) and aminoglycoside antibiotics (amikacin) exhibited low resistance rates. In contrast, penicillin antibiotics (ampicillin, piperacillin) demonstrated the highest resistance rates, exceeding 80%. As for quinolone antibiotics, which are the most frequently used drugs in this region, their resistance rates have surpassed 70% see Table 2.

**Table 2:** Antimicrobial susceptibility of 1,802 *UPEC* strains to antibiotics.

**Antibiotic**	**Susceptible N (%)**	**Intermediate N (%)**	**Resistant N (%)**
Cefotaxime	804 (44.63)	20 (1.12)	978 (54.26)
Ceftazidime	1,247 (69.19)	166 (9.19)	390 (21.62)
Cefepime	869 (48.21)	172 (9.52)	762 (42.27)
Ampicillin	210 (11.67)	7 (0.40)	1,585 (87.93)
Ampicillin/sulbactam	611 (33.88)	612 (33.94)	580 (32.17)
Piperacillin	253 (14.06)	48 (2.69)	1,500 (83.25)
Piperacillin/tazobactam	1,588 (88.14)	108 (6.01)	105 (5.85)
Amoxicillin/clavulanate	1,227 (68.09)	437 (24.24)	138 (7.67)
Amikacin	1,768 (98.10)	3 (0.17)	31 (1.73)
Levofloxacin	476 (26.44)	29 (1.60)	1,297 (71.96)
Ciprofloxacin	449 (24.94)	36 (2.02)	1,316 (73.04)
Imipenem	1,787 (99.16)	3 (0.17)	12 (0.67)
Meropenem	1,789 (99.28)	1 (0.106)	12 (0.67)

### 3.3 Detection and analysis of quinolone-related resistance genes

A total of seventy eight strains were randomly selected from 1,802 *UPEC* isolates for the detection and analysis of resistance genes. The study focused on two key genetic determinants: the QRDR genes, specifically *GyrA* and *ParC*, as well as PMQR genes, including *qnrA, qnrB, qnrD, qnrS, qepA*, and *aac (6*′*)-Ib-cr*.

#### 3.3.1 *GyrA* and *ParC* mutation sites

The amino acid sequence analysis of the *GyrA* and *ParC* mutation resistance genes in 78 *UPEC* strains isolated from urine as shown in Table 3. The *GyrA* gene exhibited a total of ten significant mutations, with the highest frequency observed at Ser83 (72 cases, 92.31%), followed by Asp87 (53 cases, 71.79%). Among the seventy eight *UPEC* strains, seventy three (93.59%) harbored mutations in the *GyrA* gene locus, including thirteen single mutations, fifty seven double mutations, two triple mutations, and one quintuple mutation. In the *ParC* gene, among the seventy eight isolates analyzed, 4 sense mutations were detected in fifty nine strains (75.64%). Of these mutated strains, 50 exhibited single mutations while nine displayed double mutations. Notably, all mutated isolates contained the Ser80Ile mutation.

**Table 3:** DNA and amino acid change of *GyrA* and *ParC* mutation.

**Gene**	**Haplotype**	**DNA change**	**Amino acid change**	**N (%)**
*GyrA*	Single mutation	C248T	Ser83Leu	12 (15.38)
		A233C	His78Pro	1 (1.28)
	Double mutation	C248T + G259A	Ser83Leu + Asp87Asn	53 (67.95)
		C248T + G259T	Ser83Leu + Asp87Tyr	3 (3.85)
		C248T + G259C	Ser83Leu + Asp87His	1 (1.28)
	Triple mutation	C248T + G259A + G156T	Ser83Leu + Asp87Asn + Met52Ile	1 (1.28)
		C248T + G259A + A262T	Ser83Leu + Asp87Asn + Thr88Ser	1 (1.28)
	Quintuple mutation	C248T + G259A + C251A + A395T+T428C	Ser83Leu + Asp87Asn + Ala84Glu + His132Leu + Val143Ala	1 (1.28)
	Wild type	-	-	5 (6.41)
*ParC*	Single mutation	G239T	Ser80Ile	50 (64.10)
	Double mutation	G239T+A251T	Ser80Ile+Glu84Val	7 (8.97)
		G239T+A251G	Ser80Ile+Glu84Gly	1 (1.28)
		G239T+C166A	Ser80Ile+Ala56Thr	1 (1.28)
	Wild type	—	—	19 (24.36)

#### 3.3.2 Analysis of PMQR gene distribution

Among the seventy eight *UPEC* strains analyzed, fifteen (19.23%) were found to carry PMQR genes. The distribution of these genes was as follows: *qnrS* was detected in seven strains (8.97%), *qnrB* was identified in four strains (5.13%), *qepA* was present in 4 strains (5.13%), and *qnrD* was observed in four strains (5.13%). Notably, *aac(6*′*)-Ib-cr* and *qnrA* were not detected in any of the tested strains. The detailed distribution of PMQR genes are presented in Table 4.

**Table 4:** Distribution of PMQR genes among *E. coli* isolates.

**Gene**	**N (%)**
*qnrB*	1 (1.28)
*aac (6′)-Ib-cr*	2 (2.56)
*qepA*	3 (3.85)
*qnrS*	6 (7.69)
*qnrB*+*qnrS*	1 (1.28)
*qnrB*+*aac (6′)-Ib-cr*	1 (1.28)
*qnrB*+*qepA*+*aac (6′)-Ib-cr*	1 (1.28)
Wild type	63 (80.77)

### 3.4 Biofilm formation ability of *UPEC*

Seventy eight *UPEC* strains were cultured in ninety six-well plates, and their biofilm formation ability was assessed using the crystal violet staining method. The results revealed that 46 strains (58.97%) were capable of forming biofilms. among these: 35 (44.87%) strains exhibited moderate biofilm formation, 11 (14.10%) strains demonstrated strong biofilm formation.

### 3.5 MIC of levofloxacin against *UPEC*

**Figure 3:**
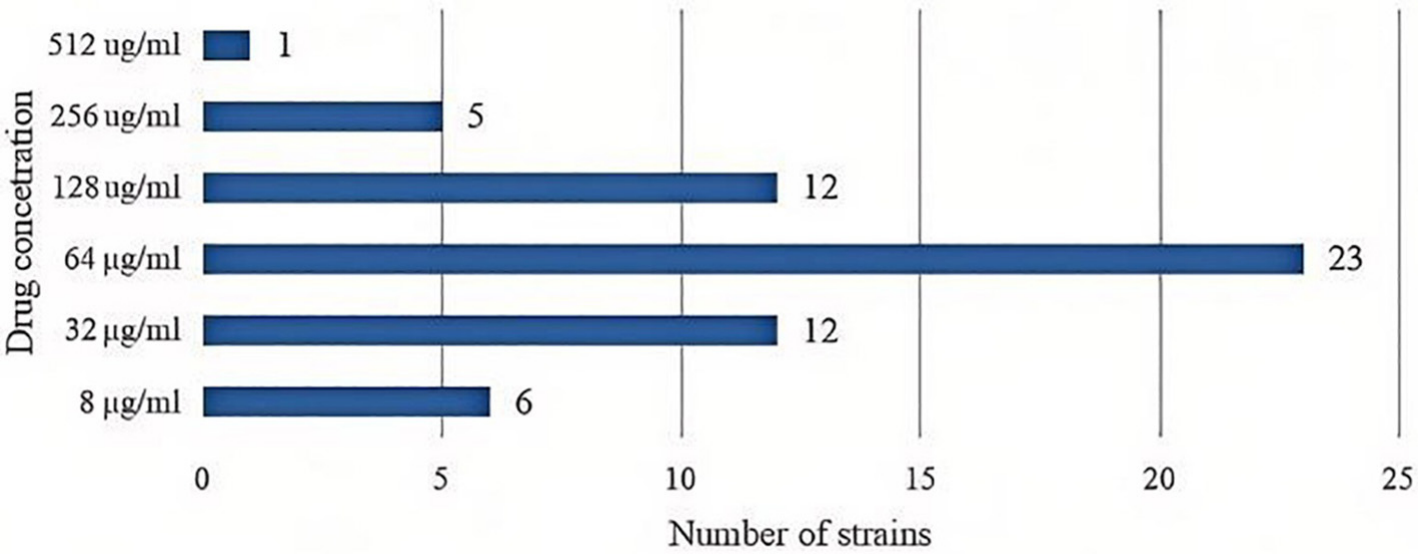
The distribution of MIC values of levofloxacin for *UPEC* strains.

Among the seventy eight *UPEC* strains, nineteen strains (24.36%) exhibited a MIC value of ≤ 4 μg/mL indicating they were sensitive to levofloxacin. In contrast, fifty nine strains (75.64%) were resistant to levofloxacin. The distribution of MIC values (see Figure 3).

### 3.6 Association analysis of *GyrA* mutants, *ParC* mutants, PMQR carriers, and phenotypic resistant strains (PRS)

**Figure 4:**
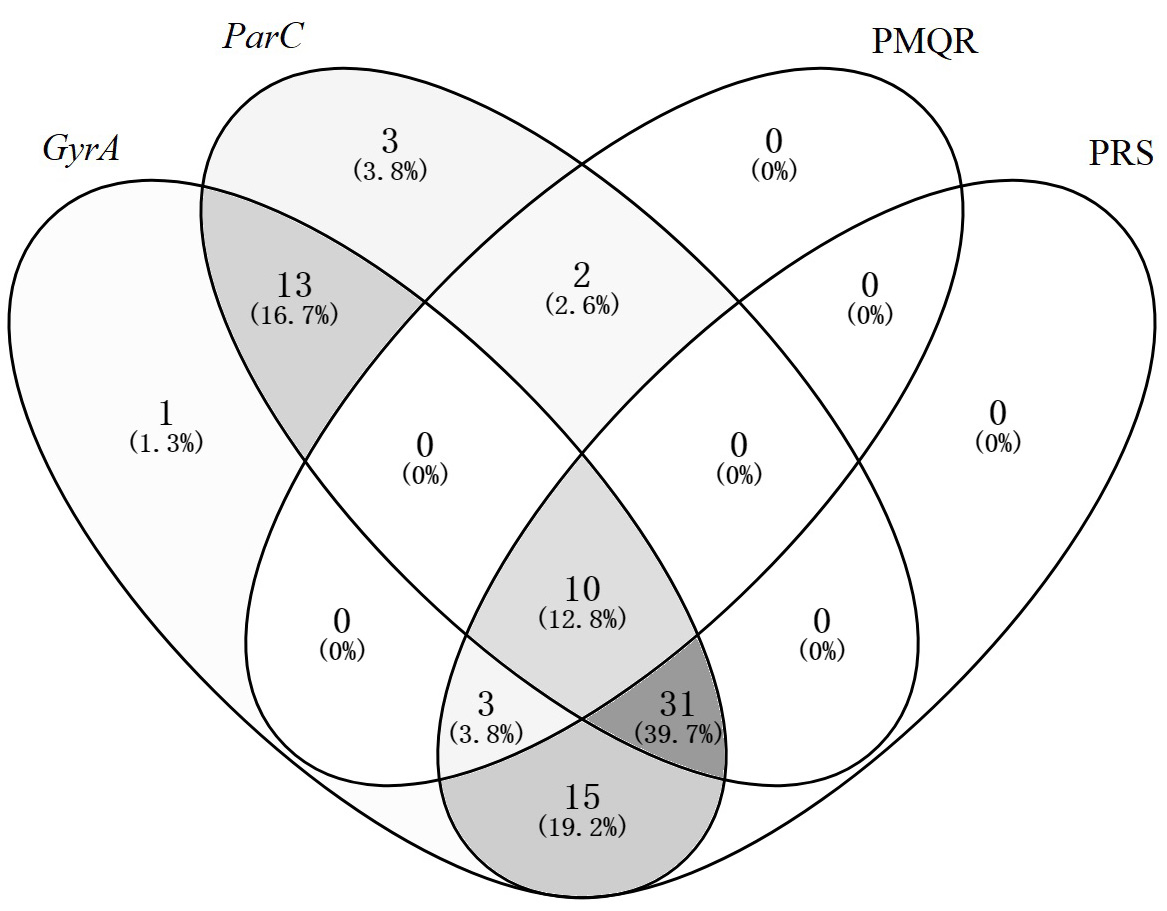
Venn diagram analysis of *GyrA* Mutants, *ParC* mutants, PMQR carriers, and phenotypic resistant strains.

Figure 4 illustrates that out of seventy eight *E. coli* strains, fifty nine (75.64%) exhibited phenotypic resistance to fluoroquinolones (FQs), seventy three (93.59%) were found to possess *GyrA* mutations, fifty nine (75.64%) had *ParC* mutations, and fifteen (19.23%) had the PMQR gene. In the PRS, *GyrA* mutation was present in all cases, with fifty nine strains exhibiting *GyrA* mutation, eighteen (30.51%)cases without *ParC* mutation, and 46 (77.97%) cases not harboring PMQR. Significantly, thirteen strains with mutations in both *GyrA* and *ParC* exhibited no fluoroquinolone resistance.

### 3.7 Molecular markers of high-level quinolone-resistance

The definition of high-level levofloxacin-resistance (HLQR) is MIC≥8μg/ml. analysis of the relationship between gene mutation sites and quinolone resistance revealed that ser83leu and asp87asn are common mutation sites in the *GyrA* gene and are closely related to quinolone resistance. as shown in Table 5: the ser83leu mutation site has high sensitivity (100%) but low specificity (42%) for resistance, while asp87asn has high sensitivity (92%) and high specificity (89%). simultaneous detection of ser83leu and asp87asn can effectively predict the resistance of clinical *UPEC* strains to quinolone antibiotics, thereby guiding clinical medication.

**Table 5:** The sensitivity and specificity of Ser83Leu and Asp87Asn in predicting HLQR.

**HLQR**	**Ser83Leu**		**Specificity**	**Sensitivity**	**Asp87Asn**		**Specificity**	**Sensitivity**
	**+**	**−**			**+**	**−**		
+	59	0	42%	100%	54	5	89%	92%
-	11	8			2	17

## 4 Discussion

*E. coli* remains the predominant pathogen associated with urinary tract infections (UTIs), as evidenced by the analysis of urine samples collected from patients at Baotou Central Hospital between 2022 and 2024. In this study, *E. coli* accounted for 19.35% of the identified uropathogens, highlighting its significant role in UTIs. However, the increasing prevalence of antibiotic resistance, particularly to fluoroquinolones, poses a substantial challenge to the effective management of these infections. The resistance rate to fluoroquinolones observed in this study (75.64%) significantly exceeds the national average(56.70%) (Fu et al., 2024), underscoring the urgency of addressing this issue in Inner Mongolia.

The mechanisms underlying fluoroquinolone resistance in *E. coli* are multifaceted. Primarily, resistance arises from mutations in the QRDR genes encoding DNA gyrase (*GyrA* and *GyrB*) and topoisomerase IV (*ParC* and *ParE*). Mutations in these genes, particularly at amino acid residues 67-106 in *GyrA* (with a notable hotspot at position 83) and residues 63-102 in *ParC*, disrupt the binding of fluoroquinolones to their target enzymes, thereby conferring resistance. Our findings align with previous studies, demonstrating that *GyrA* mutations are the primary drivers of resistance, while *ParC* mutations play a secondary role. Specifically, we identified significant mutations at Ser83 and Asp87 in *GyrA* and Ser80 and Glu84 in *ParC*, consistent with established literature (Yonezawa et al., 1995; Zhang et al., 2018; András et al., 2024; Sepideh et al., 2024). It is noteworthy that several rarely reported mutations in *GyrA* were identified in the present study: Met52Ile, Ala84Glu, His132Leu, Val143Ala, and Thr88Ser. These mutations may have potential implications for protein structure and function, warranting further investigation to elucidate their biological significance.

Moreover, this study revealed a complex mutation profile in *GyrA*, with ten distinct mutations observed across 1-5 mutation types, surpassing previous reports. The frequent co-occurrence of *GyrA* and *ParC* mutations further suggests that the accumulation of multiple mutations contributes to the elevated fluoroquinolone resistance observed in this region. Notably, all fluoroquinolone-resistant strains harbored *GyrA* mutations, whereas *ParC* mutations were not universally present, reinforcing the notion that *ParC* mutations alone cannot independently confer resistance but rather enhance resistance when combined with *GyrA* mutations.

In addition to chromosomal mutations, PMQR genes also contribute to fluoroquinolone resistance (Rehaiem et al., 2024; Samira et al., 2024). Our study detected PMQR genes, including *qepA, qnrS, qnrB*, and *aac (6*′*)-Ib-cr*, with *qnrS* exhibiting the highest detection rate. While the presence of these genes does not directly correlate with high-level resistance, they may expand the Mutation Selection Window (MSW), facilitating the emergence of chromosomal mutations under sustained antimicrobial pressure. This finding is consistent with prior research, which has documented the co-occurrence of multiple PMQR genes in resistant strains (Abimbola Olumide et al., 2022; Nabi et al., 2022; Nazek et al., 2024; Yujin et al., 2024).

The ability of *E. coli* to form biofilms further complicates its resistance profile. In this study, 58.97% of the tested strains demonstrated biofilm-forming capabilities, with 44.87% exhibiting moderate biofilm formation and 14.10% showing strong biofilm formation. Biofilms provide a protective barrier against antimicrobial agents and host immune responses (Sarah et al., 2024), thereby enhancing bacterial survival and resistance. This analytical result indicates that, under the experimental conditions of this study, there is no statistically significant (*P* > 0.05) correlation between biofilm formation capacity and bacterial resistance levels (as measured by MIC values) or the number of resistance gene mutations. This finding suggests that biofilm formation may be regulated by other independent factors, such as environmental conditions, bacterial metabolic states, or specific gene regulatory networks. This outcome provides new perspectives for a deeper understanding of the complex relationship between biofilm formation and antimicrobial resistance, while also guiding the direction of future research.

## 5 Conclusion

This study highlights the critical role of *GyrA* mutations in fluoroquinolone resistance, the synergistic effect of *GyrA* and *ParC* mutations, and the contribution of PMQR genes and biofilm formation to the resistance landscape of *E. coli*. These findings underscore the need for rational antibiotic use, enhanced resistance monitoring, and the development of novel therapeutic strategies to combat the growing threat of antibiotic-resistant UTIs.

## 6 Limitation

In this study, only seventy eight *UPEC* strains were randomly selected for quinolone resistance gene detection from 1,802 total isolates; this relatively small sample size may introduce sampling bias and reduce statistical power for validating associations between specific gene mutations and resistance phenotypes.

## Data Availability

The original contributions presented in the study are included in the article/supplementary material, further inquiries can be directed to the corresponding author.
